# Treatment Options for Anemia in Kidney Transplant Patients: A Review

**DOI:** 10.1016/j.xkme.2023.100681

**Published:** 2023-05-27

**Authors:** Mario Bonomini, Lorenzo Di Liberato, Vittorio Sirolli

**Affiliations:** Nephrology and Dialysis Unit, Department of Medicine, G. d’Annunzio University, SS. Annunziata Hospital, Chieti, Italy

**Keywords:** Anemia, kidney transplantation, iron, erythropoiesis-stimulating agent, graft failure, HIF inhibitors

## Abstract

Anemia is common after kidney transplantation. The etiology may be multifactorial, such as causes of anemia in the general population and causes that are unique to the kidney transplant setting. Posttransplant anemia, particularly when severe, may be associated with adverse effects such as graft failure, mortality, and a decline in kidney function. After careful investigation, that is, having excluded or treated reversible causes of anemia, treatment of anemia in patients with a kidney transplant is based on iron supplementation or erythropoiesis-stimulating agents (ESA), although there are no specific guidelines on anemia management in this patient population. Iron therapy is often needed, but optimal and safe iron-deficiency management strategies remain to be defined. Evidence suggests that ESAs are safe and potentially associated with favorable outcomes. Better graft function has been reported with ESA use targeting hemoglobin levels higher than those recommended in the general population with chronic kidney disease and with no apparent increased risk of cardiovascular events. These results require further investigation. Data on the use of hypoxia-inducible factor inhibitors are limited. Prevention and treatment of anemia in kidney transplantation can improve patients’ quality of life, life expectancy, allograft function, and survival.

Normocytic normochromic anemia represents a frequent complication in patients experiencing chronic kidney disease (CKD) and is associated with several adverse clinical outcomes.^1^ A successful kidney transplantation (KT) may potentially correct anemia. However, ∼20%-51% of kidney transplant recipients (KTRs) are anemic at various time points after transplantation.^2^, ^3^, ^4^ Posttransplant anemia is usually differentiated into early and late. Early anemia (≤6 months after transplantation) has a prevalence of ∼50%, whereas late anemia occurs after 6 months in 23%-35% of patients who underwent transplantation.^5^^,^^6^

Posttransplant anemia is associated with reduced exercise capacity, cognitive decline, chronic fatigue, and impairment in quality of life.^7^^,^^8^ Moreover, a growing number of observations indicate that anemia can be negatively associated with long-term clinical outcomes in KT, such as graft failure, mortality, and a decline in kidney function. Thus, it is reasonable to treat anemia in KTRs, probably starting as soon as possible after transplantation,^5^ although there are no proper or accurate guidelines for the management of anemia in this patient population. The KDIGO guidelines for KTRs^9^ and the position statement from the European Renal Best Practice Group^10^ mention treating anemia in KT by following the management guidelines for anemia in CKD.

The aim of this review was to provide an update on posttransplant anemia in adult KTRs. Anemia in pediatric KTRs has recently been described elsewhere^11^ and will not be discussed in this study.

### Causes of Posttransplant Anemia

There are many potential causes of posttransplant anemia (Box 1).

Box 1 **Main Causes of Anemia in Kidney Transplantation**

• **Early Anemia**
  ⋄ Iron deficiency
  ⋄ Immunosuppression induction
  ⋄ Infections
    ▪ Viruses causing aplastic anemia (parvovirus B19, Epstein-Barr virus, cytomegalovirus, adenovirus, BK virus, herpesviruses, and varicella-zoster virus)
    ▪ Indolent infections (bacterial, fungal, viral, and parasitic)
  ⋄ Allograft Quality
    ▪ Ischemia time
    ▪ Delayed graft function
    ▪ Extended donor criteria
  ⋄ Acute Rejection
  ⋄ Aggressive Hydration (dilutionary effect)
• **Late Anemia**
  ⋄ Allograft Dysfunction
  ⋄ Iron Deficiency
  ⋄ Immunosuppressive Treatment
  ⋄ Infections
    ▪ Late-viral (cytomegalovirus, hepatitis B virus, and hepatitis C virus)
    ▪ Community-acquired pathogens
  ⋄ Drugs
    ▪ Renin-angiotensin system inhibitors
    ▪ Proton-pump inhibitors
    ▪ Antimicrobials
  ⋄ Acute rejection
  ⋄ Vitamin B12 and Folic acid deficiency

Allograft function strongly correlates with the prevalence and severity of anemia.^12^^,^^13^ Serum erythropoietin (EPO) secretion by the graft occurs within a few days of transplantation, although it connects to effective erythropoiesis weeks later, through a second peak of serum EPO release depending on the recovery of kidney function.^14^ Serum EPO levels may fluctuate in KTRs according to the kidney function level.^15^ Serum EPO production decreases with the decline in the glomerular filtration rate (GFR) of the transplanted graft,^13^ and lower serum EPO levels are associated with poor graft function.^15^ A rapid decrease in serum EPO levels can be because of acute rejection episodes.^16^ Besides serum EPO deficiency, a serum EPO-resistant state may also be involved in the pathophysiology of posttransplant anemia, possibly related to risk factors persisting (iron deficiency, inflammation, or hyperparathyroidism) or acquired in the posttransplant milieu (infections or myelotoxic drugs).^15^

Iron deficiency is highly prevalent in KT and may contribute to both early and late anemia. Increased iron consumption because of increased serum EPO production by the allograft, and blood loss (transplant surgery, repeated sampling, use of anticoagulants, return of the menstrual cycle, and malignancies) may cause an absolute iron deficiency.^17^ On contrary, low-grade inflammation and the use of mammalian target of rapamycin (mTOR) inhibitors promote the upregulation of hepcidin, leading to reduced iron availability (functional iron deficiency) for erythropoiesis.^17^ Folate and vitamin B_12_ deficiencies can also be detected because of poor dietary intake, requiring nutritional supplementation.^18^

Several pathogens, mainly viruses, may increase the risk of anemia by direct bone marrow suppression or indirect effects such as increased inflammation causing impaired erythropoiesis.^19^^,^^20^ The time of infectious disease onset is usually in the first few months after transplant surgery, which is consistent with the stronger immunosuppression therapy during that period.^21^ However, parvovirus B19 infection, strongly suggested by a very low percentage (<1%) in the reticulocyte count, may occur as early as 2 weeks after KT.^22^

Medication-induced anemia may be considered when other causative factors are apparently absent^12^; an algorithm to manage drug-induced anemia in recipients of solid organ transplants has recently been suggested.^23^ Immunosuppressive drugs (calcineurin inhibitors, mTOR inhibitors, anti-thymocyte globulin, and antimetabolites), antivirals (ganciclovir and valganciclovir), and antimicrobials (trimethoprim-sulfamethoxazole) can, directly or indirectly, suppress the bone marrow, causing anemia.^6^^,^^23^ Several other drugs that may be used in KTRs can cause hemolytic or megaloblastic anemia.^24^ Chronic use of proton-pump inhibitors may impair intestinal iron absorption resulting in iron-deficiency anemia.^25^ Other medications associated with anemia in KTRs are renin-angiotensin system (RAS) inhibitors.^13^ After RAS activation, angiotensin II enhances erythropoiesis by increasing serum EPO secretion through tubulointerstitial ischemia,^26^ stimulating erythroid progenitors,^27^ and reducing hepcidin levels.^28^ Although the RAS contribution to erythropoiesis may be imperceptible in the general population,^29^ RAS blockade may have a hematocrit-lowering effect in patients with immunosuppression.^30^ Such an action by RAS inhibitors is exploited in the treatment of posttransplant erythrocytosis.^12^^,^^31^

### Consequences of Posttransplant Anemia

#### Decline of kidney function

Recent findings indicate that KTRs with anemia may experience a decline in estimated GFR (eGFR) over time. In a retrospective analysis comparing eGFR at 2 years with eGFR at 6 months, patients with late posttransplant anemia showed a decline of 2.26 mL/min/1.73 m^2^, whereas patients without anemia reported an increase of 3 mL/min/1.73 m^2^.^4^ The difference between the 2 groups proved statistically significant (*P* < 0.05).

Further evidence concerning an association between posttransplant anemia and eGFR decline, particularly the role of anemia correction on the decline rate, is described further in the section on ESA therapy.

#### Graft failure

A meta-analysis addressing the effect of anemia on graft survival including 11 observational studies and 11,632 KTRs reported a consistent association between anemia and poor graft outcome,^32^ as confirmed by several subsequent studies.^2^^,^^4^^,^^33^, ^34^, ^35^ During a mean follow-up time of 5.4 years, KTRs with both early (6 months) and late (2 years) anemia reported a significantly higher percentage of graft failure at 4 years than did patients without anemia.^4^ In a cohort of 1,139 KTRs (412 anemic), during a median of 5.5 year follow-up, the presence of severe anemia (hemoglobin [Hb] concentration of <11 g/dL) was significantly associated with death-censored graft failure, the association being weaker for mild anemia.^2^

A retrospective analysis of the association between graft survival and temporal changes in Hb concentration values during the first 90 days after surgery, rather than Hb concentration values at specific time points, showed that KTRs with no Hb level increase (>0.5 g/dL/mo in at least 1 interval) reported an increased risk of death-censored graft failure (the primary outcome of the study). It should be noted that neither the rate nor timing of the Hb level increase correlated with the primary outcome.^36^

#### Cardiovascular complications

Cardiovascular disease (CVD) is frequent after KT and is the leading cause of mortality.^37^ Anemia is a modifiable risk factor for CVD development in KTRs.^38^

Posttransplant anemia proved a potentially modifiable correlate of de novo congestive heart failure, which in turn was a predictor of death.^39^^,^^40^ Anemia also proved to be an independent risk factor for left ventricular growth 1-5 years after transplant, and both anemia and left ventricular hypertrophy were associated with an increased risk of mortality.^41^ An inverse association between Hb concentration and E/e’, an echocardiography index of left ventricular filling pressure, was reported in KTRs.^42^ These results suggest that, through the increase in left ventricular stiffness and pressures caused by the impairment of left ventricular relaxation, anemia may contribute to heart failure in KTRs.^42^

A recent meta-analysis and systematic review showed a significantly increased risk of CVD death and major adverse cardiovascular events in anemic KTRs when compared with those without anemia.^35^

#### Mortality

Studies examining the association of posttransplant anemia with mortality yielded conflicting results. In a prospective cohort study, at the end of the follow-up period of 4-years anemia was associated with an increased risk of mortality.^43^ A retrospective analysis showed that every increase of 1 g/dL in the Hb levels reduced the mortality by 18%.^44^ Another retrospective series reported an association between posttransplant anemia at 12 months and an increased risk of mortality.^45^ Similarly, anemia shortly after KT (3-12 months) was associated with an increased risk of mortality up to 10 years of follow-up independent of kidney function.^46^ Both early and late posttransplantation anemia (PTA) were associated with a higher mortality rate at 4 years.^4^

Other studies, however, did not confirm the association of posttransplant anemia with mortality. These include 2 retrospective studies, 1 with a follow-up of 8 years^3^ and the other involving 887 KTRs evaluated at 12 months,^47^ and a prospective cohort study with a follow-up of 5 to 6 years.^48^

A possible explanation for the discrepancies between studies examining the correlation between posttransplant anemia and mortality rate is that in studies showing no association, most patients reported mild anemia (mean Hb concentration of >11 g/dL). Thus, in a cohort study of 1,139 KTRs the association between anemia and mortality rate was related to severity. Although severe anemia (Hb concentration of <11 g/dL) proved to be strongly associated with mortality, mild anemia was not.^2^

### Treatment of Posttransplant Anemia

The approach to anemia in a KTR should begin with a diagnostic workup to search for any underlying reversible cause (Fig 1). Having excluded or treated reversible causes of anemia, the recommended treatment for posttransplant anemia is iron preparations or ESAs.

**Figure 1:**
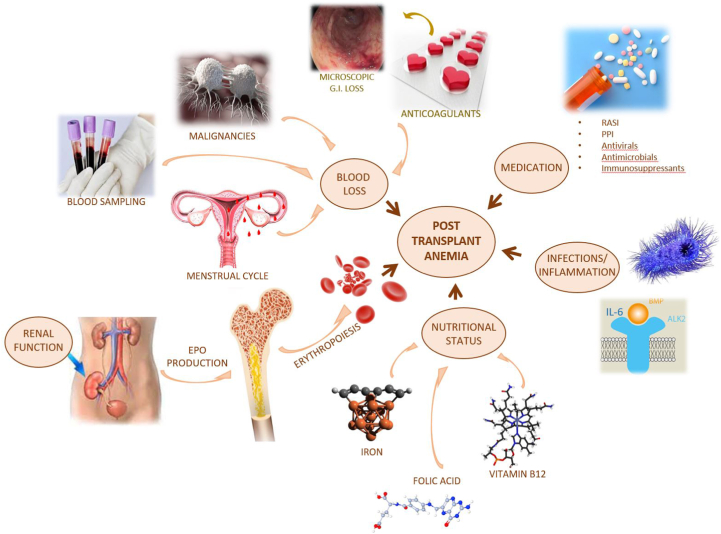
Clinical approach to evaluating anemia in a kidney transplant recipient. PPI, proton-pump inhibitors; RASI, renin-angiotensin system inhibitors.

#### Iron therapy

Data on how iron deficiency affects the clinical outcome of KT are limited. Better graft function and graft survival were associated with a higher serum ferritin level.^49^ By contrast, no association between graft failure and the percentage of hypochromic erythrocytes (an indicator of iron status) was found in the 438 KTRs.^50^ However, an independent association between the percentage of hypochromic erythrocytes and all-cause mortality was reported,^50^ whereas, in another trial, iron deficiency was independently associated with all-cause mortality even in the absence of coexisting anemia.^51^ Potential mechanisms include direct effects on cardiac and skeletal muscle metabolism. Iron deficiency may compromise cardiac and skeletal muscle cell function by decreasing intracellular oxygen availability and impairing the Krebs cycle.^17^ Furthermore, iron deficiency stimulates the expression and concomitant cleavage of fibroblast growth factor (FGF) 23.^52^ FGF23 proved an independent risk factor for graft loss, cardiovascular, and all-cause mortality in KTRs,^53^^,^^54^ likely owing to off-target effects of high FGF23 levels.^17^ Whether FGF23 may represent an intermediate between iron deficiency and adverse outcomes in KT remains to be elucidated.

There have been few studies addressing iron supplementation in KTR, and it remains unclear whether intravenous (IV) iron is better than oral iron. Oral iron preparations may be preferred for their ease of administration and low cost. However, the occurrence of gastrointestinal side effects, impaired intestinal absorption, and poor patient adherence restricts the effectiveness of oral iron. Newer oral iron agents (ferric maltol, ferric citrate, and sucrosomial iron), which are better tolerated and more effective than traditional iron salts,^55^ have not been evaluated in KTRs. However, oral iron may be harmful to the gut microbiota,^56^ and recent studies suggest that the host microbiota profile exerts an important effect on the KT outcome.^57^ Compared with oral preparations, IV iron has a higher capacity to correct iron parameters and increase Hb levels, with a similar safety profile in patients with CKD treated with dialysis or those with non–dialysis-dependent CKD.^58^ In a small retrospective study, administration of 800 mg IV iron sucrose was associated with a significant increase in Hb levels and a reduced eGFR decline.^59^ No increased risk of infection was associated with IV iron (polymaltose) when compared with oral treatment (ferrous sulfate).^60^ Newer IV iron agents (ferric carboxymaltose and ferric derisomaltose) allow for fewer infusions and are associated with reduced risk of serious hypersensitivity reactions.^55^ The safety and tolerability of IV ferric carboxymaltose have been shown.^61^ A potential concern when using IV iron polymaltose and ferric carboximaltose is that hypophosphatemia may worsen because of a sharp change in the metabolism of FGF23.^62^ This risk is particularly present in KTRs who continue to have elevated posttransplant levels after KT. However, of the 23 KTRs who received ≤1000 mg of ferric carboxymaltose, only 1 patient required short-term phosphate supplementation.^63^ Understanding the patient-related clinical effect of hypophosphatemia induced by IV iron requires further investigation.^64^

Iron supplementation may be beneficial in KTR, and IV administration may be a reasonable choice.^5^ However, several issues are still unresolved. It remains to be definitively established how far iron depletion/administration affects long-term clinically significant outcomes. The role of IV iron in conjunction with ESA administration needs to be further clarified.^65^ In addition, no effective treatment regimen to counteract disturbances of iron metabolism in KT has been devised. A proactive high-dose IV iron regimen proved to be safe and superior to a low-dose IV iron administered reactively in a recent study in patients treated with hemodialysis (HD),^66^ but no such therapeutic strategy has been investigated in the setting of KT. Thus, prospective studies are warranted to define optimal and safe iron-deficiency management strategies in KT.

#### ESA therapy

ESA therapy has revolutionized the care of anemic patients with CKD^1^ but is currently believed to be underused in KTRs.^65^ The results of randomized controlled studies (RCTs) focusing on improvement of Hb levels and graft outcome in ESA-treated KTRs are summarized in Table 1.^67^, ^68^, ^69^, ^70^, ^71^, ^72^

**Table 1:** Randomized Controlled Trials Focusing on Improvement of Hb Levels and Graft Outcome in ESA-Treated Kidney Transplant Patients

Author	Patients (n)	Intervention	Target Hb	Follow-Up	Kidney Outcome	CV Outcome	Comments
**Early PTA**							
Van Biesen et al,^67^ (2005)	ESA (n=18); control (n=22)	Subcutaneous epoetin beta (100 U/kg) 3×/wk	>12.5 g/dL	3 mo	No difference in graft function	NA	No difference in Hb but faster target Hb and fewer blood transfusions in ESA group
Nafar et al,^68^ (2012)	ESA (n=20); Control (n=20)	Subcutaneous biosimilar PD-poietin (2000 U) 3× in the first postoperative wk	NA	6 mo	Lower serum creatinine and higher creatinine clearance in ESA group	NA	No difference in Hb
Pile et al,^69^ (2020)	ESA (n=26); Control (n=27)	Subcutaneous epoetin beta	11.5-13.5 g/dL	2 y	No difference in kidney function	No increase in CV morbidity or thrombotic events	Improved vitality and mental health domains in quality of life SF-36 score in ESA group
**Late PTA**							
Choukroun et al,^70^ (2012)	Hb normalization (n=63); partial Hb correction (n=62)	Subcutaneous epoetin beta (dose steadily increased)	13.15 g/dL (Normalization group); 10.5-11.5 g/dL (partial correction group)	2 y	Reduced decline in estimated creatinine clearance, lower rate of kidney failure, higher graft survival in normalization group	No CV complications (heart failure, arrhythmia, myocardial infarction, and stroke) in normalization group; some CV events in partial correction group	Improved quality of life in normalization group
Tsujita et al,^71^ (2019)	High Hb (n=64); low Hb (n=63)	Subcutaneous or IV darbepoetin alpha or epoetin beta pegol	12.5-13.5 g/dL (High Hb group); 10.5-11.5 g/dL (low Hb group)	3 y	Slower decline of graft function in high Hb group	No cardiac disorders in high Hb group	NA
Obi et al,^72^ (2021)	High Hb target (n=74); low Hb target (n=79)	Subcutaneous methoxy polyethylene glycol epoetin beta	≥12.5 g/dL (High Hb group); <10.5 g/dL (low Hb group)	2 y	Lower decline of kidney function in high Hb group	No increased incidence of stroke in high Hb group	NA

Abbreviations: CV, cardiovascular; ESA, erythropoiesis-stimulating agent; Hb, hemoglobin; IV, intravenous; NA, not available; PTA, posttransplantation anemia.

In early posttransplant anemia, the first RCT enrolled patients with Hb concentration of <12 g/dL in the immediate posttransplant period who were randomized to subcutaneous epoetin beta thrice weekly to target Hb concentration of >12.5 g/dL, or else to placebo. No difference between the groups was observed in graft function or Hb levels at the 3-month end of the study, although the ESA group reached the Hb target level faster, showed a higher Hb level increase from the baseline level, and required fewer blood transfusions.^67^ Another RCT including patients with Hb levels between 8 and10 g/dL in the first postoperative week who were randomized to receive either 3 doses of subcutaneous EPO biosimilar or placebo showed that, after 6 months, although the Hb level was comparable between the 2 groups, lower serum creatinine and higher creatinine clearance were found in the intervention arm.^68^ More recently, KTRs experiencing anemia within 3 months posttransplant were randomized to epoetin beta targeting Hb concentrations of 11.5-13.5 g/dL or to no treatment.^69^ After 2 years, Hb concentration was significantly higher in the ESA-treated group, whereas the eGFR and rate of progression (eGFR slope) did not differ between the groups. Treatment with ESA improved some quality of life scores.^69^

In late posttransplant anemia, the Correction of Anemia and Progression of Renal Insufficiency in Transplant patients (CAPRIT) study examined the 2-year effect on graft survival and quality of life of normalizing Hb levels (target level of 13-15 g/dL) versus partial Hb level correction (10.5-11.5 g/dL) by using subcutaneous epoetin beta.^70^ The Hb normalization group showed a reduced decline in estimated creatinine clearance (2.4 vs 5.9 mL/min/1.73 m^2^ in the partial correction group), lower rate of progression to kidney failure, and higher death-censored graft survival. The quality of life also significantly improved in the full-correcting group.^70^ Tsujita et al,^71^ conducted a 3-year study in KTRs treated with either darbepoetin alpha or epoetin beta pegol (subcutaneous or IV), comparing the effect on graft function decline rate (primary efficacy end point) of sustained maintenance of high Hb levels (12.5-13-5 g/dL) as opposed to maintenance of low Hb levels (10.5-11.5 g/dL). The average decline in eGFR was significantly greater in the low Hb level group (5.1 mL/min/1.73 m^2^) than that in the group with high Hb level (1 mL/min/1.73 m^2^).^71^ More recently, 153 KTRs were randomized to either a high (≥12.5 g/dL) or a low (<10.5 g/dL) Hb target level and to either cholecalciferol or control (2 × 2 factorial design) received subcutaneous methoxy polyethylene glycol epoetin beta as ESA.^72^ Changes in the creatinine-based eGFR over a 2-year period comprised the primary outcome of the study. Among patients who completed the study period, the decline in kidney function was smaller in those with high Hb levels than in the low Hb level group (-1.6 ± 4.5 vs -4 ± 6.9 mL/min/1.73 m^2^; *P* < 0.05), with no difference between the cholecalciferol and the control group.^72^

The results of these studies suggest that in KT, anemia is associated with a decline in eGFR and that the correction of anemia may effectively reduce the rate of decline. This is in discordant with a previous meta-analysis reporting the absence of renoprotective effects by ESA in the posttransplant setting.^73^ However, major drawbacks have been identified in that study, such as heterogeneity and different primary end points.^65^

In a rat model of KT, correction of Hb level using EPO prevented histologic signs of chronic allograft nephropathy through the regulation of intragraft expression of antioxidant, antiapoptotic, and angiogenic properties.^74^ However, blood transfusions normalizing posttransplant Hb levels reported no effect on allograft injury, suggesting that the renoprotective effect of EPO outweighed the correction of anemia itself.^74^ In in vitro murine models and in a prospective clinical study in CKD stage 4 ESA-treated patients, EPO exhibited immunomodulating properties that are needed for spontaneous kidney allograft acceptance.^75^ However, early clinical studies did not show any tissue-protective benefit from using ESA in the posttransplant setting,^73^^,^^76^ which might be explained from several methodological angles.^36^ Thus, the potential renoprotective role of ESA in KT awaits better definition in adequate future studies.

Recommendations based on the level of evidence indicate that the use of ESA in early posttransplant anemia should be considered using a case-by-case approach, whereas ESA should be used in late anemia with a Hb target level between 12.5 and 13.5 g/dL to achieve better graft survival, caution being required in patients with a high risk of malignancy.^65^ This Hb target level is quite similar to the Hb target level of 12-13 g/dL recently recommended by a panel of experts.^6^ It should be noted that the recommended Hb target level in KTR is higher than the recommended target suggested in the general CKD population by KDIGO (Hb concentration of 11.5 g/dL) and KDOQI (Hb concentration of 11 g/dL) guidelines.^77^^,^^78^ The practice of targeting lower Hb levels in CKD has been guided by the undesirable results of large-scale studies regarding the safety and efficacy of ESA therapy targeting high Hb target levels in patients with CKD.^79^, ^80^, ^81^

Although results concerning cardiovascular complications at higher Hb target levels in KT need to be interpreted with caution owing to some study limitations, the evidence gathered so far in KTRs treated with ESA has not shown an increased cardiovascular risk. In the CAPRIT study, no cardiac disorders (heart failure, arrhythmia, or myocardial infarction) or stroke occurred in the full-correction Hb level group, whereas some cardiovascular events occurred in the group with a lower Hb level.^70^ Cardiovascular disorders were not noticed in the study by Tsujita et al.^71^ The study by Obi et al, showed no increased incidence of stroke in KTRs randomized to the high Hb target level arm.^72^ Moreover, no adverse cardiovascular effects or thrombotic events were observed in a 2-year trial in KTRs receiving epoetin beta with an Hb target concentration of 11.5-13.5 g/dL.^69^ The apparent discrepancies between the populations with nontransplanted CKD and KTRs may be explained by the different settings. Chronic allograft nephropathy and CKD are fundamentally different entities regarding pathophysiology and outcome.^70^ In addition, a chronic allograft kidney may prove superior to the kidney of a patient with non–dialysis-dependent CKD regarding histopathology, hemodynamics, and immune biology.^71^

The actual evidence suggests that the use of ESA in KTRs is safe and associated with a possible favorable outcome. Different types of ESA showed comparable efficacy in correcting anemia, although long-acting ESAs may be more practical in managing PTA, whereas there is no preferable way (subcutaneous or IV) for ESA administration. The optimal use of ESA from the viewpoints of dosage and Hb target level needs further clarification.

#### Blood transfusion

Red blood cell transfusion is common in KTRs, particularly in the first few months after transplant.^12^^,^^82^ However, a study including more than 12,000 KTRs reported an association between early transfusion and transplant failure defined as graft loss or death with a functional graft.^82^ Receiving transfusion within 1 month from KT was associated with reduced graft survival and an increased risk of antibody-mediated rejection and infections at 1 year.^83^ Furthermore, transfusion may have a prothrombotic effect, predisposing to venous thromboembolism.^84^ A recent retrospective cohort study described a significantly increased risk connected with receiving transfusion after transplantation: namely, venous thromboembolism, such as deep venous thrombosis or pulmonary embolism; the risk increased as the number of transfusions increased.^85^ Notably, venous thromboembolic events in KT are associated with graft loss and death.^86^

Blood transfusion cannot be considered an alternative strategy for managing posttransplant anemia because they still carry some risks, expose patients to large Hb-level fluctuations, and have limits on availability.^1^ The current evidence emphasizes the need for judicious use of transfusion in KT, weighing the risk-benefit balance, and considering other methods of anemia correction such as optimizing iron stores or ESA use.^85^

#### Hypoxia-inducible factor inhibitors

Oral hypoxia-inducible factor-prolyl hydroxylase (HIF-PH) inhibitors are now available to manage anemia in people with CKD. This novel class of EPO drugs increases the endogenous serum EPO levels, stimulates transcription of the EPO gene in kidney and hepatic tissue, and seems to reduce hepcidin levels and improve iron homeostasis.^87^ Thus, inhibition of HIF may comprehensively, and using different mechanisms, regulate the pathological factors associated with the anemia of CKD. Among HIF-PH inhibitors, roxadustat was authorized for use in patients with anemia and CKD (regardless of dialysis or not) by the European Medicines Agency in 2021, whereas daprodustat has just been approved by the US Food and Drug Administration for patients treated with dialysis for 4 months.

Data on the use of HIF-PH inhibitors in KTRs are limited. This may be mainly because of a theoretically increased cancer risk (eg, upregulation of vascular endothelial growth factor and angiogenesis), in an immune suppressed population already prone to developing malignancies.^65^ A prospective, observational study in KTRs with Hb concentrations of <11 g/dL evaluated the effect of roxadustat (20-100 mg) administered thrice weekly.^88^ The Hb target level was 11-13 g/dL, and ESAs were used if Hb levels were <10 g/dL. Of the 31 enrolled patients, 25 patients completed the study period of 20 weeks, with 7 cases of early PTA. The mean Hb levels increased progressively from 9.8 g/dL at baseline, plateauing after 20 weeks at a value of 12.4 g/dL. Iron deficiency requiring iron supplementation was observed at 8 weeks, and 12 patients received ESAs. Complications leading to drop out included reduced Hb levels in 3 patients, gastrointestinal symptoms in 2 patients, and myocardial infarction in 1 patient.^88^ Li et al^89^ examined 21 KTRs (6 ESA-treated) admitted to a hospital for complications after KT with Hb concentrations of <10 g/dL; they monitored the effect of roxadustat thrice weekly at a weight-based starting dose. Eleven patients were withdrawn from roxadustat before the study ended (10 weeks), owing to initial or later nonresponse (n = 6) or on reaching the Hb target level. In patients completing the study, the Hb concentration significantly increased from 6.9 to 10.4 g/dL, and the treatment response rate (Hb concentration increase of >1 g/dL) was 71.4%. No obvious adverse reactions were noticed.^89^ In 5 KTRs with late posttransplant anemia, who switched from epoetin beta pegol to roxadustat 100 mg thrice weekly for a 9-month period, the Hb levels increased in all patients after just 1 month, and a satisfactory improvement in anemia was maintained thereafter. However, Hb level overshooting was observed, with 1 patient suspending roxadustat after 1 month and 3 patients needing a drug dosage decrease. No serious complications were observed.^90^

From the current evidence on the use of HIF-PH inhibitors in KT, it is advisable to start with a low dose and slowly increase the titration and supplementing iron owing to increased iron utilization.^20^ RCT studies are needed to elucidate the long-term efficacy and safety of HIF-PH inhibitors in anemic KTRs.

## Conclusions

Anemia in KT needs to be carefully investigated and appropriately treated. An increasing body of evidence indicates that the presence of anemia, particularly when severe, may be associated with adverse effects on graft function and patient health. After correction of a treatable cause, iron administration and ESA use comprise the mainstay of treatment for late PTA. Correction of anemia in KTR by iron or ESA is usually well tolerated and has potential clinical benefit. HIF-PH inhibitors may represent a further therapeutic approach in the near future. However, several factors remain to be established, such as Hb target levels, proper iron therapy, and the safe use of ESAs. Resolving such issues will improve management of the anemic occurring in kidney transplant patients.
